# Buffalo Hospital of the Sisters of Charity

**Published:** 1849-07

**Authors:** F. H. Hamilton

**Affiliations:** Attending Surgeon. Buffalo


### BUFFALO MEDICAL JOURNAL

AND

### MONTHLY REVIEW.

VOL. 5. JULY, 1849. NO. 2.

### ORIGINAL COMMUNICATIONS.

ART. I.—*Buffalo Hospital of the Sisters of Charity. Surgical Department Reports of Selected Cases : by* F. H. HAMILTON, the Attending Surgeon.

*Onychia Maligna.* Joseph Comin, aged 9 years; admitted Oct. 19th, 1848. Habit scrofulous. The extremity of the great toe of the left foot was prodigiously enlarged, red, and slightly tender. The nail was black, rotten as pasteboard, and stood directly up from its matrix. This had been its condition for a year.

A bread and water poultice was applied the first twenty-four hours, and, from this time, Ung. Hyd. Rub., or the Ung. Hyd. Nit. were applied daily to the foot. The diet was generous, and he was allowed occasional exercise. Under this treatment the malady gradually disappeared; the cure being accomplished in about three months.

*Onychia Osteosa.* Ann Sullivan, aged 2 years. Entered the Hospital Sept. 24th, 1848. She is a pale, thin, feeble infant, and, at the time of her admission was suffering under a diarrhœa. Upon the last phalanx of the index finger of her right hand was an oblong, purple, and shining swelling of two weeks' existence. We opened it freely, and found the bone carious. Ten days after, having recovered from her diarrhœa, she was discharged; the finger being in nearly the same condition as when first opened. In this case, as in the *onychia maligna,* the general system was at fault.

*Acute lumbar abscess, cured.* James Knowlton, aged 23; a painter. Entered Nov. 3d, 1849. On the 21st of October he fell eleven feet, through the scuttle of the propeller Montezuma, striking upon his left side.

He got out of the hold without assistance, and continued to walk about that day and the next, with only a slight lameness in his left leg. Tuesday, the 23d, he began to be lame in his right hip; from which time the thigh has gradually become flexed upon the body, and cannot be straightened. He suffers much pain in the groin. A violent strain of the psoas muscles was diagnosed, and the occurrence of an iliac or lumbar abscess anticipated.

"℞.—Absolute diet, and moderate catharsis.

"5th.—Much pain last night. Pulse 100, feeble. Violent fever since yesterday. ℞. sulph. morph. gr. ¼ at bed time.

"Until the 12th he took regularly the morphine at bed time; occasionally a mild cathartic, and was kept upon moderate diet. On the 12th he was seized with a diarrhœa, which was checked in a few days with laudanum, and prepared chalk, and oak bark, with a better diet. From the 13th, he took, also, whenever the condition of his bowels would permit, quinine and elixir vit. in moderate doses. On the 19th fluctuation was apparent in the right groin, this announcement of the existence of pus having been preceded by a chill on the 18th.

"Nov. 22d.—Had a second chill yesterday. No night sweats; no fever; sense of oppression across the chest; slight cough. To-day opened abscess three inches below Poupart's ligament, and over the femoral artery. Discharged half a pint of pure pus and then closed the wound. Continue the quinine, grs. iv., three times daily, with three ounces of best Madeira. Good diet. Also continue the morphine at bed time.

"23d.—Last night had his first sweat. Continue treatment.

"24th.—Cough increasing, appetite gone. Abscess discharged this morning half a pint. Continue treatment.

"26th.—Symptoms about the same. Substitute f℥ iij of good brandy for the wine.

"27th.—Discharged this morning a quart of pus with flakes of lymph.

"28th.—Half a pint discharged this morning. Has pain in right side. Another chill; give five grains of quinine instead of four, three times daily, and increase the amount of brandy one ounce.

"On the 29th, iodide of iron was added to the prescription. On the 29th, 30th, and Dec. 1st and 2d, the abscess discharged about two quarts. On the 2d of Dec. it was offensive. On the third it discharged spontaneously half a pint. On the 4th he had a diarrhœa. 5th.—The matter was bloody as well as putrid—evacuated half a pint. On the 6th gas also escaped with the matter. Until Dec. 11th, each day, the pulse was more rapid and small; the emaciation increased; the cough and expectoration became more copious; the matter more abundant, offensive, bloody and mixed with gas. He seemed to be almost in the article of death, and we announced to him what seemed his inevitable fate. To this he was resigned.

"Dec. 11th.—The amount of brandy increased to three ounces three times daily, and three drops of nitric with three drops of muriatic acid substituted for the elix. vit. Quinine and iodide of iron discontinued.

From this day, and with no other change in the treatment, the improvement dates. The next morning the amount of matter was sensibly diminished, and improved in quality, and all the other symptoms were amended. On the 18th the matter had suddenly changed to pure serum, of which it discharged about two ounces. The same treatment was continued. On the 21st, after about three weeks of partial deafness, his right ear began to discharge serum also. On the 23d the serum had ceased to discharge from his ear; hearing was nearly restored; the abscess still discharged about one ounce of serum per day; cough was nearly gone; appetite good. The mineral acids were now dropped. There was no discharge from this date. On the 28th the brandy was suspended, and in about a week more he was well. The leg straightened as soon as the abscess healed. During the last two or three months he has been actively engaged in his trade, and bears no sign of disease about him. The amount of matter discharged at various times during his sickness is estimated at from ten to twelve quarts.

*Compound fracture of lower end of tibia and tarsal bones.* Patrick Murphy, aged 28 years, entered Oct. 22d, 1848. Three weeks since, his right foot was traversed by the wheel of an empty car, and badly crushed· Until received here, he had been under the care of Dr. Erastus Wallis, of this city; and the swelling, at first very great, had been considerably reduced' and the wounds partially healed. Crepitus was distinct upon slight motion of foot. A gutta percha splint was applied to leg and foot, with simple dressings to the wounds, and the whole limb placed in a Bowen's splint.

From this date until the 31st, the discharge of matter was profuse and often highly offensive. He suffered much from pain and general restlessness. Night sweats and occasional chills, also, with emaciation, indicated an approach to hectic. The treatment was chiefly tonic and stimulating. Under this plan with careful dressing and management of the leg, he gradually passed the crisis, and from about the 31st of October, more than four weeks after the accident, he slowly improved. Several fragments of bone were discharged, but when dismissed, Dec. 12th, he had tolerably free use of his ankle joint, the wounds had healed, and he had a very useful, if not perfect limb. We never absolutely condemned this limb to amputation, yet, at one time, we warned him of the probability that it would soon be necessary to make this sacrifice for the safety of his life.

*Leg torn off, amputation, and death.* Daniel Riley, aged 40. Entered Hospital Dec. 2d at 7 P. M. He had been intoxicated most of the time for a week. At 4, P. M., of the day on which he was admitted, his left leg had been completely severed just below the knee by the passage across it of a locomotive and train of cars. When he reached the Hospital he had a tournequet on the leg, above the knee. He had lost, at least, a pint of blood: no bleeding at that time; no pulse at the wrist; extremities cold; mind wandering. Under the internal use of warm stimulants and external application of heat, he had rallied considerably at 11 o'clock. Pulse 108, and distinct; mind clear; extremities warm. But the stump had commenced bleeding again pretty freely; the tourniquet had now been applied seven hours, and to tighten it more, or even to continue it as it was then applied, would endanger the vitality of the limb. Dr. James P. White, the counselling Surgeon of the Hospital, and Dr. C. C. Wyckoff, determined, with myself, that the amputation could not be delayed. I made the amputation six inches above the knee. He lost but about two ounces of blood in the operation; three arteries were tied; the muscles did not retract after they were cut. Before the wound was closed he began to sink, and in spite of stimulants, which were finally thrown into his stomach, when he became unable to swallow, by a stomach pump, he died in three hours after the operation was made.

This amputation was made before full reaction had taken place, for reasons which appear in the report, and the patient sunk under the united shock of the accident and the operation.

*Extensive injury and death.* Israel Ellsworth, first mate of schooner Harrison; aged —. Admitted Dec. 7, 1848, at 5 o'clock P. M. Five and a half hours before, he had fallen from the main mast of his vessel, forty feet to the deck, striking upon his feet, and then pitching forward upon his head. He was taken up insensible. Drs. Mixer, Newman, and White, had seen him, and employed means to restore him.

When he was admitted he was unconscious; right pupil dilated; left, contracted and dilated naturally; respiration, 36 in a minute; no stertor; pulse 164 and feeble; face pale; colliquative sweat; slight hemorrhage from left ear; moved his limbs; bladder paralyzed. There was, also, a wound on his forehead; right femur was broken twice; left tibia and fibula broken and projecting through the flesh.

Attempts were made to revive him by stimulants, &c., but he continued to sink, and died the next morning at half-past two o clock.

*Autopsy.* Effusion of blood under the scalp, and between dura mater and skull; underneath the dura matter, extensive; beneath the tunica arachnoides; around the velum interpositum; under the tentorium; around the medulla oblongata; at the bifurcation of the optic nerves; in the substance of the anterior left lobe of the cerebrum, and in the bottom of the socket of the right eye. A fracture extended through the right orbital plate of the os frontis, traversing the cribriform plate of the ethmoid, separating the lesser wing of Ingrassias from the body of the sphenoid, and crossing the petrous portion of the left temporal bone about three lines external to the meatus auditorious internus, the tympanum being entirely broken up.

In the abdomen bloody effusions existed underneath the peritoneum on every side. The spleen was ruptured at two points, and the blood poured into the cavity of the peritoneum.

The symptoms after he reached the Hospital were chiefly those of concussion; but the autopsy showed the existence of compression. The case, therefore, furnishes another illustration of the fact, that where the concussion is very great, no signs of compression can be manifested. We cannot say, because there is no symptom of compression, that compression does not exist. But this ought not to embarrass our practice, since it is still the concussion, as the predominating circumstance, which is to be regarded in the treatment.

*Supposed fracture of base of skull; recovery.* Michael McCarty; aged about 20. Entered Dec. 28th, 1848. On the 27th he was thrown from a wagon while the horses were running, and his head was traversed by one of the wheels. Three hours he remained insensible, during which time he vomited some, and bled from both of his ears and his nose; in all a quart or more. He was bled, also, twelve ounces, about four hours after the accident.

On the 28th he complained much of pain in his hands: both paralyzed. Pulse was 116, and small; respirations 28; face flushed; pupils natural; right eye turned in; partially deaf; sleep almost constant; continued to bleed from his ears; voice raucous. His hair was cut, and cold applied to his head; heat and mustard to his feet; active cathartics, followed by injections, were also administered. This plan was pursued until the 30th, when he began to move his left arm. With only slight deviations it was continued also to the 14th of January. He was then perfectly rational, and free from pain; slept well nights; had a good appetite; his left arm had recovered entirely, but his right was still paralyzed. The deafness had disappeared, but the eye still turned in.

He remained nearly three months longer under treatment. Cathartics, low diet, setons and blisters, were our remedies, and finally the use of strychnine, but he remains in nearly the same condition as he was on the 14th of January.

We believe this patient has thus far recovered from a fracture through the base of the skull, accompanied with extravasations of blood within the cranium.

BUFFALO, June, 1849.

